# Revising mtDNA haplotypes of the ancient Hungarian conquerors with next generation sequencing

**DOI:** 10.1371/journal.pone.0174886

**Published:** 2017-04-19

**Authors:** Endre Neparáczki, Klaudia Kocsy, Gábor Endre Tóth, Zoltán Maróti, Tibor Kalmár, Péter Bihari, István Nagy, György Pálfi, Erika Molnár, István Raskó, Tibor Török

**Affiliations:** 1 Department of Genetics, University of Szeged, Szeged, Hungary; 2 Department of Biological Anthropology University of Szeged, Szeged, Hungary; 3 Department of Pediatrics and Pediatric Health Center, University of Szeged, Faculty of Medicine, Szeged, Hungary; 4 SeqOmics Biotechnology Ltd., Mórahalom, Hungary; 5 Institute of Biochemistry, Biological Research Centre, Szeged, Hungary; 6 Institute of Genetics, Biological Research Centre, Szeged, Hungary; University of Florence, ITALY

## Abstract

As part of the effort to create a high resolution representative sequence database of the medieval Hungarian conquerors we have resequenced the entire mtDNA genome of 24 published ancient samples with Next Generation Sequencing, whose haplotypes had been previously determined with traditional PCR based methods. We show that PCR based methods are prone to erroneous haplotype or haplogroup determination due to ambiguous sequence reads, and many of the resequenced samples had been classified inaccurately. The SNaPshot method applied with published ancient DNA authenticity criteria is the most straightforward and cheapest PCR based approach for testing a large number of coding region SNP-s, which greatly facilitates correct haplogroup determination.

## Introduction

Comparing ancient DNA (aDNA) sequences extracted from well dated archaeological remains from different periods and locations provide crucial information about past human population history (reviewed in [1]). Phylogeographic inferences are drawn from phylogenetic and population genetic analyses of sequence variations, the quality of which can be biased by data quantity and quality. Nowadays Next Generation Sequencing technology (NGS) provides a growing number of high quality aDNA sequence data, but until recently the majority of aDNA studies have been restricted to short fragments from the hypervariable region-1 (HVR-I) of the mitochondrial DNA (mtDNA) genome, using PCR based methods. PCR based methods are very sensitive for contamination, as low amounts of exogenous DNA can easily dominate PCR products resulting in the recovery of irrelevant sequences [2–5]. As a result, in spite of the applied authenticity criteria [6], many of the published databases may contain unreliable sequences, which distort statistical analyses. This problem is especially relevant for many of the ancient populations, from which only PCR based HVR data are available.

Recently several aDNA studies were published aiming to shed light on the origin of ancient Hungarians, two of these [7,8] applied restriction fragment length polymorphism (RFLP) to identify 11 or 14 haplogroup (Hg) specific coding region SNP-s in addition to HVR sequencing, while another study [9] tested 22 coding region SNP-s with multiplex PCR and GenoCoRe22 assay described in [10].

Using the NGS method combined with hybridization enrichment, we have sequenced the entire mtDNA genome of 9 samples from the Tömöry et al. 2007 [7] study, and 15 samples from the Neparáczki et al. 2016 [9] study, so we could compare the reliability of two different traditional approaches.

## Materials and methods

### Archaeological samples

Bone samples from the Hungarian conquest period used in the study of [7] are carefully maintained in the anthropological collection at the Department of Biological Anthropology, University of Szeged, Hungary, so we could unambiguously identify and resample these remains. Bone powder remains of samples from the study of [9], were saved in the Department of Genetics, University of Szeged, and were reused to build NGS sequencing libraries.

### DNA extraction

Ancient DNA work was performed in the specialized ancient DNA (aDNA) facilities of the Department of Genetics, University of Szeged, Hungary with strict clean-room conditions. 100 mg bone powder from tooth roots, femurs or metatarsus was predigested in 1 ml 0,5 M EDTA 100 μg/ml Proteinase K for 30 minutes at 48°C, to increase the proportion of endogenous DNA [11], then DNA solubilisation was done overnight, in 1 ml extraction buffer containing 0.45 M EDTA, 250 μg/ml Proteinase K, 1% Triton X-100, and 50 mM DTT. DNA was bound to silica [12] adding 6 ml binding buffer (5,83 M GuHCl, 105 mM NaOAc, 46,8% isopropanol, 0,06% Tween-20 and 150 μl silica suspension to the 1 ml extract, and the pH was adjusted between 4–6 with HCl. After 3 hours binding at room temperature silica was pelleted, and washed twice with 80% ethanol, then DNA was eluted in 100 μl TE buffer.

### NGS library construction

First 50 μl DNA extract was subjected to partial uracil-DNA-glycosylase (UDG) treatment followed by blunt end repair, as described in [13]. DNA was then purified on MinElute column (Qiagen), and double stranded library was made as described in [14], except that all purifications were done with MinElute columns, and after adapter fill-in libraries were preamplified in 2 x 50 μl reactions containing 800 nM each of IS7 and IS8 primers, 200 μM dNTP mix, 2 mM MgCl_2_, 0,02 U/μl GoTaq G2 Hot Start Polymerase (Promega) and 1X GoTaq buffer, followed by MinElute purification. PCR conditions were 96°C 6 min, 16 cycles of 94°C 30 sec, 58°C 30 sec, 72°C 30 sec, followed by a final extension of 64°C 10 min. Libraries were eluted from the column in 50 μl 55°C EB buffer (Qiagen), and concentration was measured with Qubit (Termo Fisher Scientific). Libraries below 5 ng/μl concentration were reamplified in the same reaction for additional 5–12 cycles, depending on concentration, in order to obtain 50 μl preamplified library with a concentration between 10–50 ng/μl.

50 ng preamplified libraries were double indexed according to [15] in a 50 μl PCR reaction containing 1 x KAPA HiFi HotStart ReadyMix (Kapa Biosystems) and 1000 nM each of P5 and P7 indexing primers. PCR conditions were 98°C 3 min, 6 cycles of 98°C 20 sec, 66°C 10 sec, 72°C 15 sec followed by a final extension of 72°C 30sec. Indexed libraries were MinElute purified and their concentration was measured with Qubit, and size distribution was checked on Agilent 2200 TapeStation Genomic DNA ScreenTape.

Control libraries without UDG treatment were also made for assessing the presence of aDNA specific damages in the extract, as well as DNA free negative control libraries, to detect possible contamination during handling or present in materials.

### Mitochondrial DNA capture and sequencing

Biotinilated mtDNA baits were prepared from three overlapping long-range PCR products as described in [16], but using the following primer pairs, L14759-H06378, L10870-H14799, L06363-H10888, described in [10].

Capture was done according to [16] with the following modifications: Just four blocking oligos, given below were used in 3 μM (each) final concentration:

BO1.P5.part1F: AATGATACGGCGACCACCGAGATCTACAC-Phosphate,

BO2.P5.part2F ACACTCTTTCCCTACACGACGCTCTTCCGATCT-Phosphate,

BO4.P7.part1 R GTGACTGGAGTTCAGACGTGTGCTCTTCCGATCT-Phosphate,

BO6.P7.part2 R CAAGCAGAAGACGGCATACGAGAT-Phosphate.

For one capture 300 ng biotinilated bait was used with 30 μl Dynabeads MyOne Streptavidin C1 magnetic beads (Thermo Fisher Scientific). Double indexed libraries of 20 samples (300 ng each) were mixed and concentrated on MinElute columns, then captured together in a 64 μl hybridization reaction. When fewer samples were enriched, we used proportionally smaller amounts of baits. After washing, bead-bound enriched libraries were resuspended in 20 μl water and released from the beads in a 60 μl PCR reaction containing 1 X KAPA HiFi HotStart ReadyMix and 2000 nM each of IS5- IS6 library primers. PCR conditions were: 98°C 1 min, 10 cycles of 98°C 20 sec, 60°C 30 sec, 72°C 30 sec, followed by a final extension of 72°C 30 sec. The captured and amplified library mix was purified on MinElute column and eluted in 15 μl EB.

Before sequencing, libraries were quantified with Qubit, and quality checked and Agilent 2200 TapeStation Genomic DNA ScreenTape. Sequencing was done at the SeqOmics Biotechnology Ltd., using MiSeq sequencer with MiSeq Reagent Kit v3 (Illumina, MS-102-3003) generating 2x150bp paired-end sequences.

### Data analysis

The adapters of paired-end reads were trimmed with the cutadapt software [17] in paired end mode. Read quality was assessed with FastQC [18]. Sequences shorter than 25 nucleotide were removed from this dataset. The resulting analysis-ready reads were mapped to the GRCh37.75 human genome reference sequence using the Burrows Wheeler Aligner (BWA) v0.7.9 software [19] with the BWA mem algorithm in paired mode and default parameters. Aligning to the GRCh37.75 human reference genome that also contains the mtDNA revised Cambridge Reference Sequence (rCRS, NC_012920.1) [20] helped to avoid the forced false alignment of homologous nuclear mitochondrial sequences (NumtS) to rCRS, though the proportion of NumtS, derived from low copy nuclear genome, is expexted to be orders of magnitudes lower than mtDNA in aDNA libraries. Samtools v1.1 [21] was used for sorting and indexing BAM files. PCR duplicates were removed with Picard Tools v 1.113 [22]. Ancient DNA damage patterns were assessed using MapDamage 2.0 [23], and read quality scores were modified with the rescale option to account for post-mortem damage. Freebayes v1.02 [24] was used to identify variants and generate variant call format (VCF) files with the parameters -q 10 (exclude nucleotids with <10 phred quality) and -P 0.5 (exclude very low probability variants). Each variant call was also inspected manually. From VCF files FASTA format was generated with the Genom Analysis Tool Kit (GATK v3.5) FastaAlternateReferenceMaker walker [25].

## Results

### NGS sequencing

We have sequenced 24 complete mtDNA genomes of the ancient Hungarians with multiple coverage (Table 1) without gaps and determined the haplotypes of the individuals (Table 2 and S1 Table). For two samples (Table 1) we have replicated the experiments from two independent extracts, one from bone another from tooth derived from the same individual, and in each case received identical sequence reads. UDG treated and non UDG treated libraries derived from the same extract also gave the same sequence reads. MapDamage profile of our partial UDG treated and control non treated library molecules displayed typical aDNA damage distribution (S1 Fig), as described in [13]. MapDamage computed proportions of sequence reads with aDNA specific C-to-T and G-to-A transitions at the ends of molecules which remained after partial UDG treatment are shown in Table 1. The average length of the obtained mtDNA fragments ranged from 56 to 85 bp (Table 1), an expected size range for aDNA [26]. These data indicated that the majority of sequences were derived from endogenous DNA molecules. Then we have estimated the percentage of possible contaminating molecules (Table 1) with a similar logic as in [27], by calculating the proportion of reads which did not correspond with the diagnostic positions of the consensus sequence given in S1 Table, which revealed very low contamination levels. Phylogenetic analyses (HaploGrep 2, [28]) of all consensus sequences resulted unambigous classifications without contradictory positions. Consensus sequences were submitted to NCBI GenBank under Accession No: KY083702-KY083725.

**Table 1:** Details of NGS data for each sample.

cemetery/grave no. /sample name	sample source	total no. of reads	no. of reads mapped on rCRS	no. of unique mapped reads	average fragment length	Average coverage	(%) of nucleotides above 5x coverage	estimated contamination (%)	(%) G to A misincorp. at 3' end (MapDamage)	(%) C to T misincorp. at 5' end (MapDamage)
Magyarhomoróg/120/anc2	tooth	26152	12519	2994	55.97	10.2	89.62	0.00	6.11	7.81
Orosháza-Görbics tanya/2/ anc3	femur	85178	50555	5516	79.37	24.8	99.89	0.43	8.13	8.89
Szabadkígyós-Pálliget/7/anc4	tooth	53176	27002	5006	63.85	19.3	97.46	0.45	8.19	9.22
Szegvár-Oromdülő/412/anc5	tooth femur	242925	139136	74712	68.45	302.9	100.00	1.47	15.11	16.15
Szegvár-Oromdülő/593/anc6	tooth	66798	35260	6488	58.09	22.8	98.64	2.08	6.47	8.70
Sárrétudvari-Hízóföld/5/anc10	tooth	17632	6664	4284	69.94	17.9	95.11	0.00	7.40	10.29
Sárrétudvari-Hízóföld/118/anc12	tooth	36214	14708	6218	63.96	24.0	98.81	0.36	8.75	11.53
Sárrétudvari-Hízóföld/213/anc13	tooth	42326	20383	9424	62.44	34.2	95.01	0.83	9.01	10.22
Harta-Freifelt/10/anc25	tooth	135472	66169	7938	80.75	35.8	99.93	1.75	6.00	7.10
Karos-III/1	femur	30830	5968	4738	81.44	22.4	98.07	0.00	13.09	11.48
Karos-III/3	femur	58982	12858	7414	76.84	32.0	98.91	1.68	12.51	10.66
Karos-III/4	femur	82194	22930	9316	68.71	38.3	99.98	0.33	12.93	11.98
Karos-III/5	metatarsus	60797	25043	20236	85.13	100.2	100.00	3.76	7.99	6.65
Karos-III/6	femur	41054	3863	1886	70.68	7.9	87.24	0.00	6.71	6.75
Karos-III/8	femur	75724	26747	11426	64.26	43.9	99.67	1.79	11.37	11.08
Karos-III/10	femur	67416	6371	3438	68.22	14.1	89.61	0.00	9.89	9.04
Karos-III/11	femur tooth	203927	75860	57508	69.95	240.7	100.00	1.43	13.48	15.45
Karos-III/12	femur	52738	5843	4742	70.76	20.0	95.80	3.96	12.40	11.19
Karos-III/14	femur	61346	16134	8778	69.29	35.7	99.69	1.82	14.53	14.13
Karos-III/15	femur	142977	83486	24702	81.12	115.4	100.00	0.22	9.78	9.39
Karos-III/16	femur	90950	23233	5334	75.65	23.6	99.14	1.34	11.34	11.12
Karos-III/17	femur	43330	2626	2382	79.64	11.2	87.55	0.00	10.31	9.91
Karos-III/18	femur	9184	3208	3154	68.48	12.9	90.49	0.00	15.42	12.11
Karos-III/19	tooth	59102	30135	5948	69.07	24.6	98.78	0.00	6.39	6.55

Data refer to paired-end sequences from UDG treated libraries. The Szegvár-Oromdülő/412/anc5 and Karos-III/11 samples were sequenced twice from tooth and femur with identical results, then these sequence reads were merged, and statistics are given for the merged reads.

**Table 2:** Comparison of Haplogroups identified with different PCR based methods and NGS.

	cemetery/grave no. / sample name	HVR-I muations found (position -16000)	HVR-II and coding region mutations studied / method	HVR-II and coding region mutations found	Hg described in the sudy (Hg with Haplogrep)	Haplotype identified by NGS in the present study	unnoticed SNP-s, or *erroneously identified SNP*-*s* in the region studied
**Tömöry et al. 2007 samples**	Magyarhomoróg/120/ anc2	CRS	73 7028 14766 / RFLP	-	H (H2a2a1)	H84	none
	Orosháza-Görbics tanya/2/ anc3	147A 172C 183C 189C 223T 320T 355T	10238 / RFLP	10238C	N1a (L3e2b)	N1a1a1a1a	none
	Szabadkígyós-Pálliget/7/ anc4	223T 356C	10400 12308 12705 / sequencing	12308G	U4 (U4a2b)	N1a1a1a1	16147A 16172C 16248T 16320T 16355T ***16356C*** 10398G ***12308G*** 12705T
	Szegvár-Oromdülő/412/ anc5	CRS	73 7028 14766 / RFLP	14766T	H (R0)	K1c1d	16224C 16311C 73G 7028T
	Szegvár-Oromdülő/593/ anc6	114A 192T 256T 270T 294T	12308 / sequencing	12308G	U5a1 (U5a2a)	U5a2a1b	none
	Sárrétudvari-Hízóföld/5/ anc10	129A 148T 223T	10238 / RFLP 12705 / sequencing	10238C 12705T	I (N1)	I5a1a	16391A
	Sárrétudvari-Hízóföld/118/ anc12	126C 182C 183C 189C 294T 296T 298C	9 bp del / elecrtophoresis	9 bp del*	T (T2f1a)	T2f1a1	none
	Sárrétudvari-Hízóföld/213/ anc13	311C	73 14766 / RFLP 11719 12308 12705 / sequencing	73G 11719A 14766T	R (R1)	J1c3g	16069T 16126C ***16311C***
	Harta-Freifelt/10/ anc25	294T 304C	73 7028 14766 / RFLP 10310 / sequencing	-	H (H5a4)	H5e1a	none
**Neparáczki et al. 2016 samples**	Karos-III/1	183C 189C 217C	HVR-II: nt.190-309 sequenced and coding region 22 SNP-s of the GenoCoRe22 assay determined in all cases	263G 7028T 9bp del 11719A 14766T	B4	B4d1	207A
	Karos-III/3	362C		239C 263G	H6	H6a1b	none
	Karos-III/4	069T 092C 126C 261T		228A 263G 295T 7028T 11719A 12612G 14766T	J1c7	J1c7a	none
	Karos-III/5	183C 189C 217C		263G 7028T 9bp del 11719A 14766T	B4	B4d1	none
	Karos-III/6	189C		263G 7028T 9bp del 11719A 14766T	B4’5	B4d1	16183C, 16217C
	Karos-III/8	051G 189C 362C		263G 7028T 11467G 11719A 14766T	U2e	U2e1b	217C, 16129C, 16256T,
	Karos-III/10	304C		263G	H5	H5e1	16189C 16294T
	Karos-III/11	189C 223T 278T		195C 257G 263G 6371T 7028T 11719A 12705T 14766T	X2f	X2f	16093C
	Karos-III/12	183C 189C 223T 290T 319A		235G 263G 4248C 7028T 11719A 12705T 14766T	A	A12	none
	Karos-III/14	126C 163G 186T 189C 294T		195G 263G 7028T 11719A 13368A 14766T	T1a	T1a1b	none
	Karos-III/15	069T 126C 362C		263G 295T 7028T 11719A 12612G 14766T	J	J2a1	16263 del
	Karos-III/16	256T 270T		263G 7028T 11467G 11719A 14766T	U5a	U5a1a2a	16399G
	Karos-III/17	362C		239C 263G	H6	H6a1a	none
	Karos-III/18	126C 163G 186T 189C 294T		214G 263G 7028T 11719A 13368A 14766T	T1a10a	T1a10a	none
	Karos-III/19	126C 163G 186T 189C 294T		214G 263G 7028T 11719A 13368A 14766T	T1a10a	T1a10a	none

Hg-s determined incorrectly with PCR methods are hightlighted with pink background, while yellow bakground hightlights correct Hg-s with incorrect haplotypes. Erroneously identified SNP-s are labelled with bold italic and lined through. Haplogroups and haplotypes were determined with the HaploGrep 2 version 2.1.0 [28] based on Phylotree 17 [39] from the available SNP-s. For the [7] samples HaploGrep assignment, based on their identified SNP positions is given in parenthesis.

*data from Ph.D thesis of Tömöry 2008.

In NGS sequence reads typical aDNA sequence alterations, present in individual molecules, are disclosed and excluded by averaging multiple reads. Moreover aDNA specific sequence alterations, primarily C-T and G-A transitions accumulating at the end of molecules, serve as markers to distinguish ancient molecules from contaminating modern DNA. Therefore NGS eliminates most sequencing uncertainties inherent in PCR based methods (reviewed in [29]), resulting in very reliable sequence reads. So we could use our NGS data to reevaluate and compare previous haplotyping strategies used in [7–9]. For this end, from our NGS data, we collected all SNP-s within the HVR stretches and coding region positions, which had been examined in [7] and [9], then contrasted these with the original dataset (Table 2).

### Contrasting NGS and PCR based sequence data

We found that in [7] haplotypes of 5 out of 9 samples were determined correctly, while in one sample haplogroup was correct with inaccurate haplotype, and in 3 samples NGS detected entirely different haplogroups. In the 15 samples of [9] the same haplogroups were assigned from NGS data in all cases, however only 8 haplotypes proved to be correct. In both studies the majority of deviations originated from undetected SNP-s in sequencing reactions of PCR fragments, but [7] also identified 3 SNP-s erroneously (lined through nucleotide positions in Table 2). These results indicate that haplotypes from both studies were rather unreliable, but haplogroup classification with the approach of [9] is more trustworthy than with approach used in [7].

## Discussion

As multicopy mtDNA is best preserved in archaeological remains than low copy nuclear DNA, most ancient sequences are derived from mitochondria [30]. Within mtDNA, the most polymorphic HVR control region contains outstanding phylogenetic information, therefore HVR sequencing has been the primary method of choice for mtDNA hapolotyping. However HVR polymorphisms have a limited reliability for haplogroup determination, therefore in addition several informative coding region SNP-s (CR-SNP) were selected to unambiguously define haplogroups [31]. At the beginning individual CR-SNP-s were determined with RFLP [32] or direct sequencing of PCR clones, but soon multiplex PCR combined with the SNaPshot technique [33] offered a more straightforward solution for identifying multiple SNP-s simultaneously. Latter method was soon adapted in the ancient DNA field [34] [10].

Determining individual CR-SNP-s separately is very time consuming and expensive, so it is tempting to test just those CR-SNP-s which are in line with HVR-I data. This is exactly what we read in [7]: *“In cases when haplogroup categorization was not possible on the basis of HVSI motifs alone*, *analysis of the diagnostic polymorphic sites in the HVSII region and mtDNA coding region was also performed*.*”* A major problem with this approach is the ambiguity of sequence reads derived from aDNA PCR clones, as amplification typically starts from a mixture of endogenous and contaminating human DNA molecules [3]. Erroneous HVR reading will lead to inappropriate CR-SNP selection, and in case of dubious CR-SNP results, false Hg classification. This is the most probable explanation of the 3 incorrectly defined haplogroups in [7] (Table 2). A major advantage of the GonoCore22 SNaPshot assay is that all Hg specific CR-SNP-s are examined irrespectively of HVR reads. The 22 CR-SNP alleles independently define a certain Hg, which must correspond with that based on HVR sequence. As both HVR and CR-SNP reads may give ambiguous results, this approach provides a double control for correct Hg designation, but is not immune against incorrect HVR haplotype reads. This is the explanation of correct Hg-s and erroneous haplotypes in [9] (Table 2).

**Fig 1:**
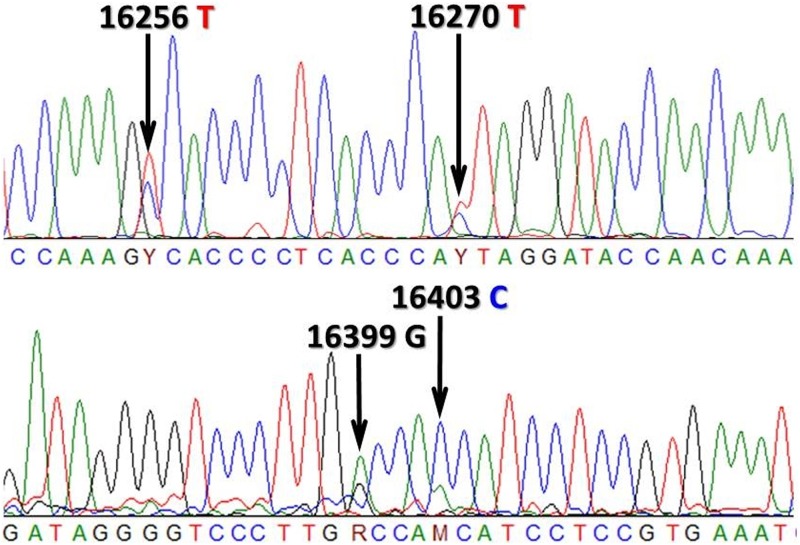
Chromatogram of two HVR-I sequence fragments of the Karos-III/16 sample from [9]. Arrows label double peaks, correct reads according to NGS data are listed above the arrows.

The problem of ambiguous aDNA sequence reads is demonstrated on Fig 1. In [9] consequently the higher peaks were taken into account, which also matched with the GenoCoRe22 data. However in position 16399 the correct nucleotide is defined by the neglected lower peak (G instead of A, see Table 2), which resulted in incorrect haplotyping. In contrast in the neighboring double peak (16403 in Fig 1), the correct nucleotide is defined by the selected higher peak.

**Fig 2:**
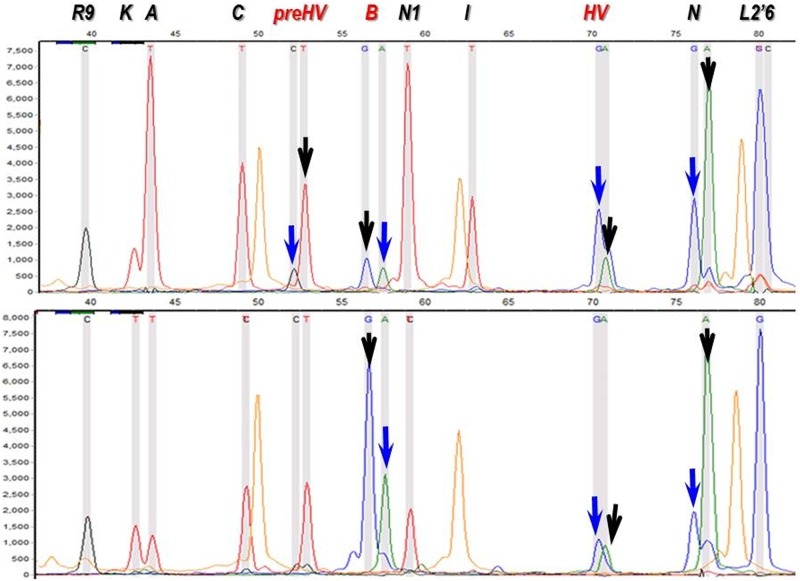
Electropherograms of two SNaPshot SBE-II reactions from two extracts of the same Karos-III/6 sample [9]. Characters at the top indicate Hg-s defined by the corresponding peaks. Black characters indicate peaks defining the ancestral allele, read characters indicate peaks defining the derived allele. Arrows point at double peaks. As each dye has a different influence on DNA mobility, positions of identical fragments with different dyes are not the same. Black arrows point at peaks taken into account, while blue arrows indicate neglected peaks, considered to have been derived from contamination. Orange peaks are size standards (GeneScan-120 LIZ, Applied Biosystems).

Coding region SNP testing with either RFLP, sequencing or SNaPshot method also suffers from the same problem as demonstrated on Fig 2. After multiplex PCR amplification of 22 mtDNA fragments two separate Single Base Extension (SBE) reactions are performed, and each reveals 11 Hg defining alleles. Both independent SBE reactions shown in Fig 2 contain several double peaks, and one of each must have derived from contamination. Some of these can be excluded from repeated SNaPshot reactions, for example the lower electropherogram excludes the ancestral *preHV* allele, since it has a single peak (T) in this position. If such exclusion is not possible, the higher peaks are preferably chosen, as the blue peak (G) for Hg *B* and the green (A) for Hg *N* on Fig 2. These decisions however must be handled with caution, therefore the presence of the *B* Hg defining 9 bp deletion also had been confirmed in [9], with singleplex PCR and agarose gelelectrophoresis. In other cases phylogenetic relations are taken into account [35], for example if the *preHV* allele is derived the *HV* allele must also be derived, this is why we have considered the lower peak (A) for *HV* in Fig 2 [9]. The summary of repeated SNaPshot reactions considered together with multiple HVR sequence reads warrants trustable Hg classification.

The studied conqueror samples were excavated between the 1930-90s, and had been handled by a large number of researchers, many with untraceable identity. It follows that these samples were inevitably contaminated during sample collection and storage. Tömöry et al. 2007 [7] collected samples from a large number of cemeteries, and published the ones with best DNA preservation. In spite of careful sampling their available method was error prone. Neparáczki et al. 2016 [9] aimed at characterizing an entire cemetery which limited the ability of sample selection, so in spite of the more reliable method their haplotype determination proved error prone. The lesson from this study is that PCR based haplotypes need to be handled cautiously, which has been well known in the aDNA field [2] [36–38]. It also follows that incorrect haplotypes particularly distort sequence based statistical analysis, like Fst statistics or shared haplotype analysis applied in [7,8]. The accumulation of authentical NGS ancient DNA sequence data in databases will greatly facilitate reliable population genetic studies.

## Supporting information

S1 Table Mitochondrial sequence haplotypes of the 24 ancient samples. SNPs are provided against rCRS. Following the recommendations in [40], we excluded common indels (hotspots) at nucleotide positions: 309.1C(C), 315.1C, 523-524del (or 522-523del), 3106del, 16182C, 16183C, 16193.1C(C), 16519C.
(XLSX)

S1 Fig Damage patterns of libraries generated by MapDamage 2.0 [23]. **a**. non UDG treated library shownig C to T (and complementary G to A) misincorporations at the 5’ and 3’ termini of the last 25 nucleotides. **b**. Damage pattern of partial UDG treated library derived from the same extract. As expected the nontreated library contains much higher rate of transitions, most of which was removed by partial UDG treatment. Only data from one extract are shown, as all libraries displayed similar pattern.
(TIF)
