# Supplementary figures and images for: Revising mtDNA haplotypes of the ancient Hungarian conquerors with next generation sequencing
Source: PLoS One. 2017 Apr 19;12(4):e0174886. doi: 10.1371/journal.pone.0174886 (PMC5396865; doi:10.1371/journal.pone.0174886)

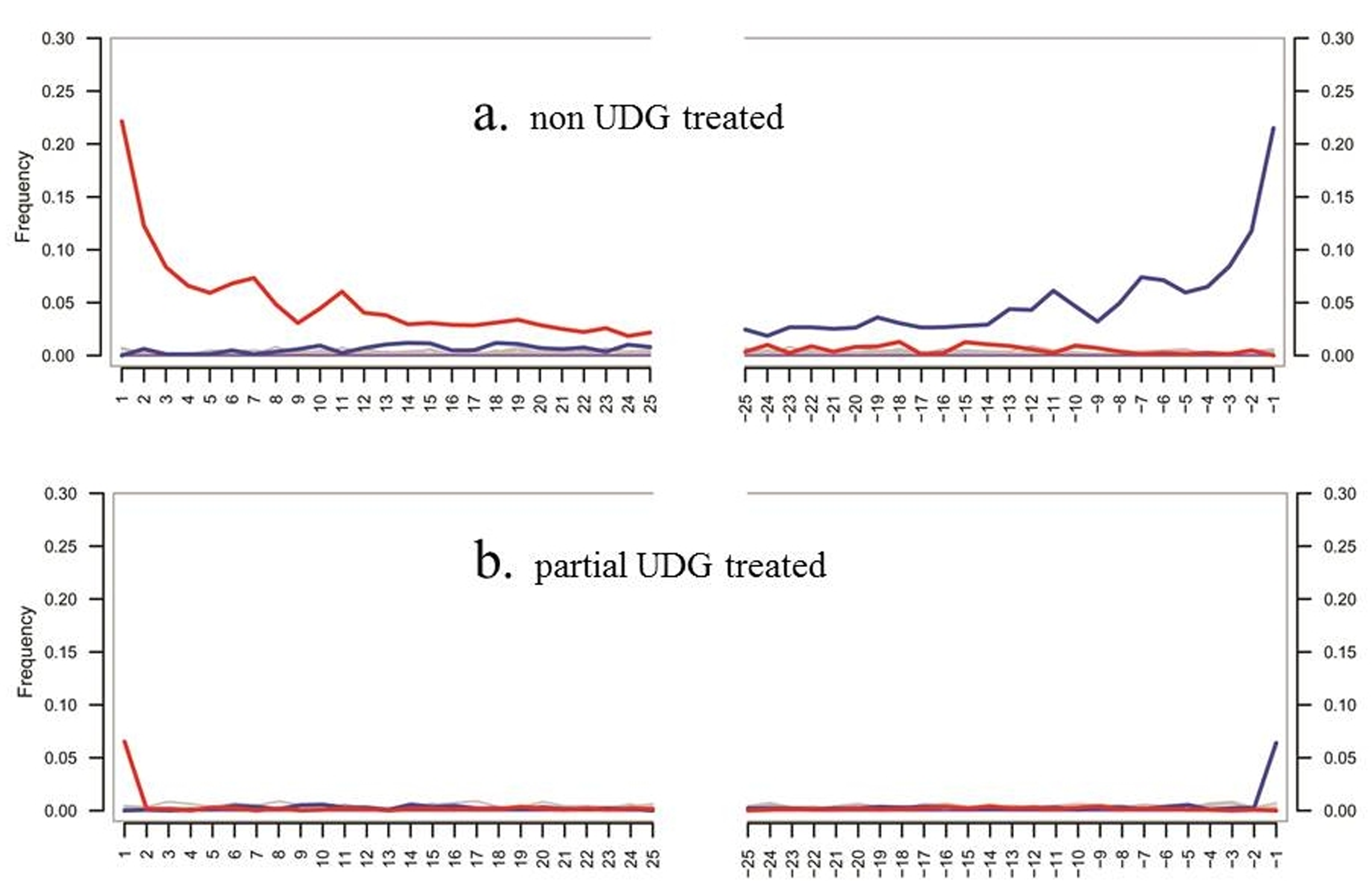

Supplement: S1 Fig — a. non UDG treated library shownig C to T (and complementary G to A) misincorporations at the 5’ and 3’ termini of the last 25 nucleotides. b. Damage pattern of partial UDG treated library derived from the same extract. As expected the nontreated library contains much higher rate of transitions, most of which was removed by partial UDG treatment. Only data from one extract are shown, as all libraries displayed similar pattern. (TIF) [file pone.0174886.s002.tif]
